# Oral Dysbiosis in Pancreatic Cancer and Liver Cirrhosis: A Review of the Literature

**DOI:** 10.3390/biomedicines6040115

**Published:** 2018-12-11

**Authors:** Hiba Mohammed, Elena Maria Varoni, Andrea Cochis, Massimo Cordaro, Patrizia Gallenzi, Romeo Patini, Edoardo Staderini, Carlo Lajolo, Lia Rimondini, Vincenzo Rocchetti

**Affiliations:** 1 Department of Health Sciences, Università del Piemonte Orientale UPO, 28100 Novara, Italy; hiba.mohammed@med.uniupo.it (H.M.); andrea.cochis@med.uniupo.it (A.C.); vincenzo.rocchetti@med.uniupo.it (V.R.); 2 Fondazione Novara Sviluppo, 28100 Novara, Italy; 3 Department of Biomedical, Surgical and Dental Sciences, Università degli Studi di Milano, 20142 Milano, Italy; elena.varoni@unimi.it; 4 Interdisciplinary Research Center of Autoimmune Diseases (IRCAD), 28100 Novara, Italy; 5 Institute of Dentistry and Maxillofacial Surgery, Fondazione Policlinico Universitario A. Gemelli, IRCCS, Roma, Università Cattolica del Sacro Cuore, 00168 Rome, Italy; Massimo.cordaro@unicatt.it (M.C.); patrizia.gallenzi@unicatt.it (P.G.); romeo.patini@unicatt.it (R.P.); edoardo.staderini@unicatt.it (E.S.); carlo.lajolo@unicatt.it (C.L.)

**Keywords:** oral microbiota, dysbiosis, inflammatory mediators, pancreatic cancer, liver cirrhosis, risk, probiotics

## Abstract

The human body is naturally colonized by a huge number of different commensal microbial species, in a relatively stable equilibrium. When this microbial community undergoes dysbiosis at any part of the body, it interacts with the innate immune system and results in a poor health status, locally or systemically. Research studies show that bacteria are capable of significantly influencing specific cells of the immune system, resulting in many diseases, including a neoplastic response. Amongst the multiple different types of diseases, pancreatic cancer and liver cirrhosis were significantly considered in this paper, as they are major fatal diseases. Recently, these two diseases were shown to be associated with increased or decreased numbers of certain oral bacterial species. These findings open the way for a broader perception and more specific investigative studies, to better understand the possible future treatment and prevention. This review aims to describe the correlation between oral dysbiosis and both pancreatic cancer and liver cirrhotic diseases, as well as demonstrating the possible diagnostic and treatment modalities, relying on the oral microbiota, itself, as prospective, simple, applicable non-invasive approaches to patients, by focusing on the state of the art. PubMed was electronically searched, using the following key words: “oral microbiota” and “pancreatic cancer” (PC), “liver cirrhosis”, “systemic involvement”, and “inflammatory mediators”. Oral dysbiosis is a common problem related to poor oral or systemic health conditions. Oral pathogens can disseminate to distant body organs via the local, oral blood circulation, or pass through the gastrointestinal tract and enter the systemic circulation. Once oral pathogens reach an organ, they modify the immune response and stimulate the release of the inflammatory mediators, this results in a disease. Recent studies have reported a correlation between oral dysbiosis and the increased risk of pancreatic and liver diseases and provided evidence of the presence of oral pathogens in diseased organs. The profound impact that microbial communities have on human health, provides a wide domain towards precisely investigating and clearly understanding the mechanism of many diseases, including cancer. Oral microbiota is an essential contributor to health status and imbalance in this community was correlated to oral and systemic diseases. The presence of elevated numbers of certain oral bacteria, particularly *P. gingivalis*, as well as elevated levels of blood serum antibodies, against this bacterial species, was associated with a higher risk of pancreatic cancer and liver cirrhosis incidence. Attempts are increasingly directed towards investigating the composition of oral microbiome as a simple diagnostic approach in multiple diseases, including pancreatic and liver pathosis. Moreover, treatment efforts are concerned in the recruitment of microbiota, for remedial purposes of the aforementioned and other different diseases. Further investigation is required to confirm and clarify the role of oral microbiota in enhancing pancreatic and liver diseases. Improving the treatment modalities requires an exertion of more effort, especially, concerning the microbiome engineering and oral microbiota transplantation.

## 1. Introduction

The oral microbiota refers to a highly varied and complicated ecosystem of bacteria, fungi, viruses, and archaea. These organisms naturally colonize the healthy oral cavity [1,2] in a relatively stable equilibrium [3] that includes more than seven hundred diverse bacterial species, one hundred fungal species, as well as a large viral population [4]. These microorganisms inhabit the ecological niches of both hard and soft oral tissues, in the form of biofilms [5]. Diverse microbial species prefer to inhabit specific niches with differing surface textures and functions [6,7].

Bacteria are generally classified within five main phyla: (i) Firmicutes, (ii) Bacteroidetes, (iii) Actinobacteria, (iv) Proteobacteria, and finally (v) Verrucomicrobia [8]. Prevalent oral groups include *Streptococcus, Neisseria, Veillonella, Actinomyces*, and other obligate anaerobes [6]. Oral bacteria include aerobic, anaerobic or facultative anaerobic bacteria with the *Fusobacterium*, *Veillonella*, and the *Streptococci* genera [6], as well as a plethora of species, not-yet cultivated and identified by means of the next generation sequencing technologies.

While the role of oral bacteria in human health and disease is increasingly well-characterized, the role of oral virome and mycobiome, remain largely uncharacterized [2,4]. The most common oral fungi are the Candida species that causes oral candidiasis when it undergoes dysbiosis. The common oral viruses include Herpes viruses, such as the herpes simplex virus 1 (HSV-1), the *Cytomegalovirus* (CMV), and the Epstein-Barr virus (EBV), and have been suggested to be associated with periodontitis [2,4].

The human body harbors distinct microbiomes, in different areas, that are substantial to homeostasis preservation. As long as these microorganisms maintain equilibrium at the appropriate site and function, they benefit the body systems. The microbiota’s benefits include support during digestion, the synthesis of vitamins B and K, prevention against pathogenic colonization [9,10,11,12], and a better outcome of immunotherapy [13]. Much evidence demonstrates the oral microbes’ role in balancing health conditions, including immune response, carcinogenesis, metabolic activity, and nutrient digestion [13,14,15].

Microbial equilibrium is preserved by the host’s immune system, which inhibits invasion of these organisms within the local tissue [9,10,11]. Within the oral cavity, microbial balance is maintained by several mechanisms that reduce their concentrations. These include the constant shedding of epithelial lining cells and salivary secretions as an example of immunoglobulins IgM, IgG, and IgA. Furthermore, agglutinins contained in the saliva, histatins, lactoferrins, and lysozymes prevent microbial insult [5,16].

Moreover, the majority of oral herpes viruses are bacteriophages contributing to the maintenance of a bacterial balance. Most of twenty-eight phages are part of the *Caudovirus* families: *Siphoviridae* (recognized as lysogenic), *Myoviridae* (sometimes lytic), and *Podoviridae* (mostly lytic) [2,17]. However, it should be considered that the virus communities belonging to the oral cavity can largely change in relation to the host sex [18].

Conversely, saliva is also a substantial source of proteins, glycoproteins, and other nutrients that preserve microbial ecology [5]. 

Increased or decreased bacterial levels act as biomarkers of healthy/unhealthy microbial performance [19]. Speaking about oral viral communities, it is well-known that they can cause a severe immune reaction in the host. Accordingly, it can be speculated that they have a crucial role in the preservation of oral immunity and in the rise of diseases [2,18,20].

## 2. Gut Microbiota and Immune Response in Cancer

Novel researches detect a major interactive network, through which the host gut bacteria interact with the immune cells result in a good or bad health status [21].

Recent studies argue that this microbial-immune network correlates gut bacteria with the whole-body health and the failure of immune homeostasis manifest significant impact on various diseases, which might result in cancer [22,23,24].

Indeed, the neoplasm consequence is not exclusively dependent on the host genotype but the immune system efficiency also plays a significant role. The immune system’s role in cancer was explained by previous studies that were carried out by applying enteropathogenic bacteria like *Helicobacter hepaticus* (*H. hepaticus*) on the immune-deficient mice that lack functional T and B lymphocytes. These experimental studies revealed both the carcinogenic incidence/suppression induced via microbe-innate immune system interaction [24,25,26].

Amongst the whole immune cells, neutrophils manifest a particular influence, as they play a key role in the development and growth of cancer [27,28].

Clinical investigative statistics show a correlation between high blood (neutrophil:lymphocyte) ratio and poor cancer prognosis, including a higher possibility of metastatic tendency [29,30]. In addition, many studies have demonstrated that poor neoplastic prognosis is related to the existence of neutrophils in the tumor microenvironment [29,30,31] and animal models have revealed the neutrophil contributions in the different stages of cancer [29,31,32]. This might be attributed to the crucial role of neutrophils in boosting tumor growth and metastasis [29]. One of the different modalities of neoplasm enhancement is achieved via releasing cytokines, such as IL1-β, IL-17, and IL-23 by the tumor inflammatory environment, as well as the tumor cells. Therefore, up-regulation of the granulocyte colony stimulating factor (G-CSF) takes place. Accordingly, neutrophils are produced and mobilized from the bone marrow, into the peripheral blood circulation, as an urgent demand. It has been observed that during expansion of the mature/immature neutrophils, T-cell proliferation is suppressed by the so-called myeloid-derived suppressor cells (MDSCs) and, thereby, the anti-tumoral influence of the T-cells becomes annulled [21,33,34,35]. Moreover, when neutrophils infiltrate a tumor, these tumor-associated neutrophils (TANs) shape the tumoral microenvironment by releasing growth factors, chemokines, and serine proteases, which encourage tumoral motility, migration, invasion, and expansion. These metastatic events are further supported by the neutrophils’ role in facilitating the tumoral cell intravasation, adhesion in the endothelium, and extravasation [29,32,36].

## 3. Systemic Involvement by Oral Bacteria

Dysbiosis refers to poor microbial adaptation or imbalance, on or inside the body [8], which results in a poor health status. Since the oral cavity is considered to be the leading entryway to the human body, the microbiota that reside within this ecological niche are very likely to expand to the various body areas [37]. When oral bacteria undergo dysbiosis, they become pathogenic and can damage the oral mucous membrane and use it as a passageway to reach the blood stream [38]. Whether oral bacteria migrate and colonize distant diseased body-sites or the same clonal origin are normally found in other body-sites, remains unclear. Regarding the potential dissemination of oral bacteria, throughout the body, some researchers have suggested bacteria continuously migrate via swallowing or the blood circulation system, within the oral cavity [39,40,41]. Thereafter, systemically these pathogens generate exaggerated quantities of the inflammatory mediators, and modify the body’s immune response. The inflammatory mediators released by the immune system include activated complement product C3a, interleukin 6 (IL-6), and the acute-phase reactant secretory phospholipase A_2_ (sPLA_2_) [42,43] Consequently, this process can promote certain systemic diseases [44], including cardiovascular diseases, diabetes, metabolic syndrome [5], and organ abscesses at distant sites [38,45].

Along these lines, recent studies have supposed a correlation, via bacterial dissemination, between oral dysbiosis and a risk of both pancreatic cancer (PC) and liver disease.

### 3.1. Pancreatic Cancer (PC)

PC is a major fatal malignant disease that occupies the fourth ranking [46] among cancer-associated mortalities, worldwide [46]. The disease’s high fatality is attributed to difficulties associated with early diagnosis and shortages in efficient therapy, stemming from drug resistant neoplasms. In addition, PC has the propensity to vigorously spread and metastasize to regional lymph nodes and distant organs. Each of these factors limit the five-year survival rate to less than 5% [46].

Many well-known risk factors contribute to PC—alcohol consumption, chronic pancreatitis, genetic mutations, environmental hazards, and cigarette smoking. Cigarette smoking serves as the major cause for pancreatic adenocarcinoma—approximately 25% cases are attributed to cigarette smoking. Increased risk of PC also relates to long-term type 2 diabetes mellitus present for more than 10 years. Furthermore, recent onset of diabetes might be an initial indicator of PC. Many previous studies have reported associations between increased PC-risk and increased body mass index (BMI) [47]. Other studies state that persons with cirrhosis appear to be at higher risk of PC [48].

#### 3.1.1. Pathophysiology of PC

Chronic pancreatitis, an inflammatory disease that precedes PC, is characterized by progressive fibrotic destruction of the pancreatic secretory parenchyma. Regardless of the disease’s diverse risk factors, pathogenic processes commonly include inflammation, pancreatic duct obstruction or necrosis/apoptosis. These cumulative processes subsequently distort pancreatic lobular morphology, modify the islet arrangement, and deform pancreatic ducts. These irreversible structural alterations impair both endocrine and exocrine pancreatic functions, and ultimately, result in neoplasm. Generally, disease prevalence largely reflects culture and geography [49].

#### 3.1.2. Association between PC and Oral Dysbiosis

Oral environmental balance plays a crucial role in oral health, and when oral dysbiosis occurs, dental diseases can result, such as dental caries and periodontal disease.

Many recent epidemiological investigations have identified an increased PC-risk in association with poor oral health conditions [50,51,52,53,54]. Since oral health status is strictly affected by the oral microbial equilibrium and activities, researchers have hypothesized that this association between PC-risk and oral health conditions might stem from the oral bacterial status [55,56]. In support of this hypothesis, a previous study has demonstrated the presence of elevated levels of blood serum antibodies for select oral pathogens—namely, *Porphyromonas gingivalis*—that were accompanied with a two-fold higher risk of PC [56,57].

To address this concern, direct assessment of oral bacterial samples was carried out using high-throughput genomic sequencing. Results revealed that specific gram negative oral bacteria are associated with a three-fold increased risk of PC. The study also identified these bacteria in the human pancreatic duct. Similarly, recent cohort studies have reported that individuals with greater circulating antibodies, for multiple oral bacteria, are at greater risk of PC [57]. In a 2013 study conducted by Michaud et al., the levels of ATTC 53978 antibodies against *P. gingivalis* were found to be higher in the four hundred and five patients with PC, as compared to the healthy volunteers [58].

Although direct correlation between PC and oral bacteria has not yet been established, a patient with a history of periodontal disease associated with circulating antibodies, against specific oral pathogenic bacteria, was shown to be accompanied by an increased risk of PC. *P. gingivalis* and *Aggregatibacter actinomycetemcomitans* are well-known keystone oral pathogens that initiate periodontal diseases and a subsequent tooth loss [59,60,61]. Some studies have argued that this condition is accompanied by an increased risk of PC [50,51,52,53,54].

PC-risk increases with the carriage of the periodontal pathogens *P. gingivalis* and *A. actinomycetemcomitans*. Previous research has found that *P. gingivalis* can invade the host immune system and disrupt signaling pathways, via cytokine and receptor degradation [62]. Furthermore, researchers have recently demonstrated that both *P. gingivalis* and *A. actinomycetemcomitans* are capable of initiating Toll-like receptor (TLR) signaling pathways, and that TLR activation is a crucial promoter of PC in animal models [57,62].

One previous study conducted by Barton et al. demonstrated that PC patients are mostly affected by a specific mutation in the cell cycle controller p53 that is related to the loss of an arginine [63].

A possible explanation comes from a recent study that showed how the bacterial peptidyl arginine deaminase (PAD) enzyme can be responsible for this mutation, when present in PC patients. *Porphyromonas gingivalis, Prevotella intermedia, Tannerella forsythia, and Treponema denticola* have been shown to hold the PAD enzyme; its activity can be linked to the modification of the Pro allele p53Arg72-Pro that is considered to be a risk factor in PCPC cases [64,65].

On the other hand, greater multitudes of the *Fusobacterium* and its genus *Leptotrichia* are associated with a lower risk of PC. However, the other *Fusobacteria* genus (*Alloprevotella*) might be accompanied by PC-risk [62]. As an opportunistic pathogen, Leptotrichia is responsible for many infectious diseases [62,66,67], including periodontitis, osteomyelitis, bacteremia, pneumonia, lung abscess, and endocarditis. These bacteria elicit an immune response that results in elevated levels of serum antibodies for this bacterial type [66]. Some have suggested that protection against PC is associated with the immune response elicited by these bacteria [62].

The abundance of various oral bacteria and their relation to PC-risk is summarized in Table 1.

#### 3.1.3. Presence of Oral Bacteria within the Pancreas

An investigative study for patients with calcific pancreatitis was conducted by Swidsinski et al. [68], using fluorescence in situ hybridization. They revealed the presence of dense multispecies bacterial biofilm comprised of different types of oral bacteria, in the pancreatic duct. These bacterial species were also found in the brains of Alzheimer’s patients [69], distal esophageal tissue [70], atherosclerotic plaques [71,72], and the feto-placental unit [73]. Whether oral bacteria migrate and colonize distant diseased body sites or the same clonal origin are normally found in other body sites, remains unclear. Regarding the potential dissemination of oral bacteria throughout the body, some researchers have suggested bacteria continuously migrate, via swallowing, or the blood circulation system within the oral cavity [39,40,41].

Further investigative study is required to clearly identify this correlation and clarify the immune system’s role in PC.

### 3.2. Liver Cirrhosis

Liver cirrhosis is the histological transformation of hepatic tissue developed by the regenerative nodular tissue formation. This nodular tissue becomes surrounded by fibrous bands, as a result of the chronic hepatic injury that lasts for several years or decades and, consequently, causes portal hypertension and end-stage liver disease [74,75]. It is one of the most widespread health conditions related to morbidity and resulting in mortality [74].

Several diverse etiological factors contribute to liver cirrhosis. The most predominant include viral hepatitis, alcoholic liver disease (ALD), and nonalcoholic fatty liver disease/nonalcoholic steatohepatitis (NAFLD/NASH) [76,77]. Recent studies have presented some evidence of a possible relationship between liver diseases and dysbiosis of oral microbiota [78,79,80,81,82,83,84,85].

#### 3.2.1. Pathophysiology of Liver Cirrhosis

Regardless of etiologic factors, liver pathosis is primarily triggered by inflammation that produces disease-specific inflammatory mediators and causes hepatocellular changes.

Liver cirrhotic transformation results when the upregulation of these mediators excites hepatocytes, hepatic stellate cells (HSCs), Kupffer cells, cholangiocytes, and hepatic T cells, and trigger the production of inflammatory molecules, like TNF, TGF-β1, and IL-6. Once the HSCs are stimulated by these inflammatory mediators, they begin to produce myofibroblasts, which in turn develop collagen and result in fibrosis and cirrhosis, over time [86,87].

#### 3.2.2. Association between Liver Disease and Oral Dysbiosis

While the correlation between liver and periodontal diseases has not yet been proven [88], clinical cases demonstrate an association between periodontitis and some types of liver pathosis, including cirrhosis, hepatocellular carcinoma, and pre-cirrhotic NAFLD [80]. Aberg and colleagues presented a relation between the Periodontal disease and chronic liver disease progression, suggesting that oral disease plays a role in cirrhosis [81].

Another study conducted by Yoneda et al. reveals that *P. gingivalis* (one of the most common periodontal pathogens) infection was mostly observed in the NAFLD positive patients, rather than the NAFLD negative patients [82]. In addition, Yoneda et al. demonstrated through an in vivo mice model that the *P. gingivalis* infection was effective in promoting NAFLD progression towards NASH. Moreover, Nagao et al. inspected the correlation within periodontal diseases and liver fibrosis advancement in hepatitis B- and C-related cirrhosis [83]. In fact, they demonstrated that the *P. gingivalis* fimbrillin A genotype was strongly present in the saliva of patients affected by liver cirrhosis [81,84,85].

Their research stated that periodontal diseases might be accompanied by a viral liver disease progression [83]. After considering various cirrhosis etiologies, periodontitis predominance was reported as higher among alcoholic cirrhosis patients [81,84,85].

#### 3.2.3. Mechanism of the Oral Bacteria Dissemination and Liver Affection

An investigative study of oral microbial functionality documented how endotoxin levels as well as their ability to fold with protein, were much higher in cirrhotic patients rather than healthy ones.

This may indicate the oral-gut-liver axis as the main reason of the inflammation cascade activation load in cirrhotic patients [79]. When intestinal permeability is impaired, the gut could likely act as a hallway between the oral cavity and the liver; bacteria metabolites and inflammation activators could be directly transmitted to the systemic blood circulation [88]. Bacteria and their products could then access the liver through the portal vein and react with the innate sensors (TLRs and NLRs) of hepatocytes and Kupffer cells, inducing inflammatory mediator release and consequently causing the cirrhosis development [89,90]. A prospective study conducted by Qin et al., matched fecal bacteria of patients with liver cirrhosis with healthy control participants. The investigation revealed increased levels of *Streptococcus* and *Veillonella* species from the feces of patients affected by cirrhosis [91]. Accordingly, the authors concluded that oral bacteria likely disseminate within or invade the gut, due to modifications in intestinal pH or bile acid (BA) dysregulation associated with cirrhosis. Moreover, cirrhosis is associated with gastric acid and bile secretion impairment, which facilitates the oral microbes’ translocation within the gut. Another study focused on investigating oral bacterial extension to the liver, during the administration of omeprazole, a proton pump inhibitor (PPI) that decreases stomach digestive acid secretion. When the gastric pH was raised, bacteria were able to pass without being killed [92].

## 4. Diagnosis of PC and Liver Cirrhosis by Oral Microbiota Status

There is a well-established, tight relationship between oral and general systemic health conditions [93]. Some studies posit that oral microbial dysbiosis may act as an indicator of the disturbed health conditions [94,95], and microbial alteration has been shown to accompany the increased PC risk, chronic inflammatory liver diseases, and hepatocellular carcinoma [96] The role of the microbiota may be of help in diagnosis, prognosis, and therapy, and clinical investigations are increasingly rely on oral microbial analysis, also, for systemic disease diagnosis [93]. Oral microbiome investigation is most commonly based on the 16S ribosomal RNA (rRNA) gene sequencing. Tongue coat investigations have revealed noticeably higher levels of *Oribacterium* and *Fusobacterium*, in liver cirrhotic patients, compared to healthy controls [96,97]. While salivary investigative studies have shown increased *Enterobacteriaceae* and *Enterococcacea* in cirrhotic patients [98]. Examinations of mouth wash samples have demonstrated that an increased abundance of *P. gingivalis* and *A. actinomycetemcomitans*, associated with a decreased relative abundance of Fusobacterium and its genus *Leptotrichia*, were subsequently related to an increased PC-risk [62]. These findings might provide prospective non-invasive diagnostic biomarkers in cirrhotic and pancreatic diseased patients [96].

## 5. Treatment Efforts

### 5.1. Engineering and Harnessing the Microbiome

Microbiome-based therapeutics, prepared to ameliorate human health by altering the associated microbial communities, could utilize modulatory, additive, or subtractive techniques. Modulatory therapies encompass modifying the activity or composition of the endogenous microbiota, by administering prebiotics or nonliving agents [99]. Additive therapies supply the microbiota with engineered or natural microorganisms [100,101,102,103], given either, individually, or as groups of strains. Subtractive therapies aim at altering the host interactions by removing certain microbiome members. In the not so-distant future, both subtractive and additive means might be utilized, jointly, to attain a larger effectiveness on the microbiome [104].

#### 5.1.1. Additive Approaches

Considerable health advantages have been accredited to natural, human-associated microbes. *Lactobacillus* spp., *E. coli*, and *Bifidobacterium* spp. have the prospect to cure a diversity of illness [105,106,107,108] which, indeed, can be found in over the-counter probiotics [104].

##### Probiotics Therapy

Probiotics can be defined as living microorganisms that provide health-beneficial roles, when properly administered to the host [109]. These bacteria primarily fall within the lactic acid bacteria [LAB] category, such as *Lactobacillus* spp. and *Bifidobacterium* spp. [110]. They are generally ingested with fermented foods or administered as dietary supplements. Several researchers have provided evidence that among gut microbiota, probiotics play a prospective role in preventing cancer development at all stages.

Interestingly, the pancreas does not host a particular microbiome; nevertheless, it is extensively influenced by gut dysbiosis. As mentioned above, bacterial endotoxins and antigens pass through the portal blood and stimulate pancreatic macrophages, to release inflammatory cytokines, like interleukins and tumor necrosis factors in necrotic pancreatic tissues. Inflammatory cytokines are one of the principle causes of chronic pancreatitis and subsequent pancreatic carcinogenesis. As a result, commensal microbiota, such as probiotics, have been increasingly utilized for both PC-prevention and treatment. Scientific studies have demonstrated that gut microflora roles are positively-affected by probiotics, which are therapeutically influenced via the immune system stimulation and modulation of the indigenous intestinal microflora composition and metabolic activity. This impact in turn prevents bacterial overgrowth and inhibits pathogen colonization [111]. Other studies have proposed strong evidence that not only supports the preventive roles of probiotics—including LAB—but also confirms their implicit ability to bind or metabolize the mutagenic carcinogens [112,113].

Other investigative studies have revealed one probiotic’s ability to alleviate fungal mycotoxins and toxic heavy metals, which may act as potential pancreatic oncogenic factors [114,115,116,117]. These investigations were performed by involving *Propionibacterium*, a well-known dairy probiotic. Thus, PC risk could potentially be lowered by decreasing the bioavailability of these neoplastic components [118].

Evidence demonstrates probiotics’ influences on the innate microbiome in non-cirrhotic patients [119]; nevertheless, further investigation is required to determine an influence of probiotics among cirrhotic patients.

A randomized controlled trial (RCT) demonstrated that when volunteers received a dose of *Lactobacillus rhamnosus* GG (LGG), twice a day, via oral administration for an eight weeks total, the level of Enterobacteriaceae was noticeably reduced while, on the opposite, the autochthonous *Clostridiales incertae sedis* XIV and *Lachnospiraceae* were increased in the feces [120]. These evidences were ascribed as a loss of dysbiosis; in fact, after eight weeks of LGG administration, the level of endotoxin and inflammation markers (such as TNF-α) was reduced [120]. These findings led to the consideration of the hypothesis of probiotics being promising candidates for endotoxemia treatments.

Probiotics also affect the heterocyclic aromatic amines (HCA), which, once exposed to high temperature, become converted to active derivatives that induce tumorigenic mutations. This process primarily relates to the ingestion of meat stewed at high temperatures. Related studies concluded that binding or disintegration of HCA, by probiotics, may be one principle mechanism for eliminating these oncogenic factors from the body [118].

##### Engineered Microbes Therapy

The recombinant expression of therapeutic biomolecules, from engineered microbes, perhaps raise these benefits and assist in the alleviation of inflammation, inhibit infection, and cure metabolic disorders. Bacteria may develop to deliver drugs at the location of the illness, boosting bioavailability and minimizing drug inactivation. Moreover, the aforementioned bacteria may be equipped with sensors which reveal disease biomarkers and trigger on-demand drug release. Completely independent, “smart” cell-based therapeutics, aimed at restoring the health of a human host have not yet been developed, clinically, but the essential technologies are accessible. The main defiance in creating microbiota-based therapeutics is the pinpointing and customization of bacterial communities to treat complex human illnesses, in spite of the variety of human-associated microbiota [104].

One implementation of engineered bacteria is to treat bacterial and viral infections. The normal flora existing in healthy people is able to withstand host colonization by pathogens, and cellular engineering can increase such withstanding [104].

Hyperammonemia is a further metabolic provision, for which engineering the microbiota might be established effectively. In the gut, bacterial ureases convert urea made by the liver to ammonia (NH3) and carbon dioxide (CO2). Hyperammonemia take place when excessive ammonia assembles systemically and give rise to neurotoxicity and encephalopathy, in the liver-diseased individuals. In mouse models, reconstituting the microbiota modified the community-wide urea metabolism [121]. After depletion of the endogenous microbiota and transplantation of a defined microbial community that manifest low urease activity, it was shown that the urease levels remained stable for months [121]. The redefined microbiota enhanced survival and decreased cognitive defects linked with hyperammonemia, in a hepatic injury model. Consequently, modifying an existing microbial community may protect against, in opposition to metabolic diseases. Moreover, microbes have been genetically-engineered to degrade ammonia and are shown to decrease the levels of systemic ammonia, while being fed to mice, like those therapies that are presently being developed by firms, for clinical attempts [122,123]. Other uses of normal flora cellular engineering are summarized in Table 2.

#### 5.1.2. Subtractive Approaches

Subtractive therapies attempt to remove harmful members of the microbiome, utilizing approaches like antibiotics, chemicals, peptides, and bacteriophages [104].

##### Antibiotic Therapy

Antibiotics, a key example of the subtractive therapies that show effectiveness on the human microbiome. Antibiotic rifaximin’s influence on the microbiome has been deeply detailed. In fact, a recent study demonstrated that an eight weeks oral administration (twice/day) of 550 mg rifaximin, in patients affected by cirrhosis, caused a reduction in the microbiota community shift, settlement, and metabolism [124,125]. Several scientific studies have suggested rifaximin’s ability to modify microbiota and, thereby, reducing inflammation, as its administration has been documented to improve life quality in cirrhotic patients [126,127].

Unfortunately, antibiotics frequently have the unfavorable influence of killing a wide set of microbes outside of the desired target. This can lead to diverse side-effects, like increased susceptibility to pathogenic bacteria, comprising the *Clostridium difficile* [104]. In the not so distance future, subtractive therapies for the microbiome should be much more specified in therapeutic activity.

##### Phage Therapy

One approach for quite specified subtractive therapies utilizes phages, which, are natural viral parasites-infecting bacteria, frequently killing the bacterial host, in the production of the phage progeny operation. The growing threat of antibiotic-resistant pathogens has rekindled attention in phage therapy [127,128], particularly taking into consideration that phages, frequently, particularly attack, exclusively one or a few cell types of bacteria, and, consequently, can be applied as more targeted antimicrobials [104].

Alongside using natural phage isolates, phages can be adjusted to hold additional or alternative functions to broaden their uses. Immunoglobulin-like protein domains on the capsids of specific phages’ exterior, increase the association with mucus [129], a mechanism that may possibly be utilized to localize phage to certain parts of the body or to broaden residence time in the gut. Host range might be reprogrammed to change the bacterial targets [130] and genes can be introduced to ameliorate the biofilms bacteria killing outcome [131]. Moreover, phages have been utilized to transport DNA to bacteria that reverses the antibiotic resistance [132,133] or to achieve nonspecific [134] or sequence-specific [135,136] antimicrobial activity to the targeted cells. Recently developed tools like CRISPR-Cas [137] genome editing and construction approaches, involving Gibson [138] and yeast [130] assembly, will expedite the not so-distance future engineering attempts. Phages, as therapeutics for microbiota-associated diseases, act as a hopeful and encouraging field of exploration and utilizing them as tools to modify the microbial communities may enable the systematic probing of the aforementioned populations for innovation and justifiability, in both the health investigation and the microbiome [104].

### 5.2. Oral Microbiota Transplant

It is well-known that periodontitis is closely-related to a dysbiosis of the microbial consortia, driven by environmental changes, such as a protein-rich/neutral-to-weakly alkaline-pH environments [7,8,9].

In periodontitis, certain members of the microbial community can destabilize the host immune response, which might result in the destruction of the periodontal tissues, in susceptible individuals. Conventional therapies for periodontitis aim at controlling the formation and metabolic activities of the supra- and subgingival biofilms [139]. Oral microbiota transplant (OMT) has been hypothetically proposed by few dental researchers.

Authors suggested an OMT procedure consisting of: (1) Collecting sub- and supra-gingival plaque from a healthy donor (spouse or a partner), (2) performing deep cleaning, root planning, and applying a broad-spectrum antimicrobial agent to the periodontitis patient, and finally (3) Neutralizing the antimicrobial agent, immediately, followed by a rinsing with a microbial suspension, harvested from the healthy donor in the periodontitis patient [140].

Despite the scanty scientific and clinical evidence, oral microbiota transplant has held promise as a new therapy for managing periodontitis, as well as caries, and some associated systemic conditions. This is because recent epidemiological investigations have identified an increased PC-risk in association with poor oral health conditions [50,51,52,53,54], accompanied by elevated levels of blood serum antibodies for select oral pathogens—namely, *P. gingivalis* [56] as well as higher levels of genotype fimbrillin *P. gingivalis*—in the saliva of patients affected by cirrhosis [83]; OMT however is not exempted by limitations, as safety might not have been secured. The transplanted biofilm should be of a high genetic-stability and should not transmit infection or induce disease. Although this technique is still theoretical, the potential adverse effects concerning those associated with fecal microbiota transplantation (FMT), could be taken into account; these effects may include transmission of infection/pathogen or induction of a chronic disease by potential microbial alteration [141].

OMT may represent a cost-effective approach and have the ability to better reach difficult to access high-risk populations. However, clinical recommendations for the use of OMT cannot be provided at this point, on the basis of the current state of knowledge. It is crucial to have a better understanding of the retentiveness of the transplanted oral biofilms, while maintaining the natural balance of the resident oral microbiota, with the host immune responses. The already understood issues include optimal donor selection, sample preparation, vehicles, follow-up timing, and the number of administrations [139].

## Figures and Tables

**Table 1:** Common oral bacteria and their associated pancreatic cancer risk.

Bacteria	Pancreatic Cancer Risk
*P. gingivalis*	Increased risk
*A. actinomycetemcomitans*	Increased risk
*Tannerella forsythia*	Not associated
*Prevotella intermedia*	Not associated
Fusobacteria	
Genus *Leptotrichia*	Decreased risk
Genus *Alloprevotella*	Increased risk

**Table 2:** Genetically-engineered normal bacterial flora and their possible therapeutic applications.

Genetically Modified Bacteria	Role	In Vivo Model
Probiotic *E. coli nissle*	Prophylaxis: inhibition of *Vibrio cholera* virulence.	Mouse
*Lactobacillus jensenii*	Prevent transmission of chimeric simian/ human immunodeficiency virus (SHIV).	Rhesus macaque
*Lactococcus lactis*	Suppress pro-inflammatory cytokine secretion: by secretion of recombinant interleukin-10 (IL-10). Treatment of autoimmune diabetes: by further modification of this IL-10 secreting *L. lactis* to produce auto-antigen proinsulin or glutamic acid decarboxylase-65.	Mouse
*Lactococcus lactis*	Treatment of oral mucositis: by secretion of trefoil factor-1.	Hamster
Probiotic *E. coli*	Reduction of obesity: via synthesizing anorexigenic lipids precursors.	Mouse
*Lactobacillus gasseri*	As glucagon-like peptide (GLP-1) delivery vehicle: to induce conversion of intestinal epithelial cells into insulin-producing cells.	Rat

## Abbreviations

IgA	Immunoglobulin A
IgG	Immunoglobulin G
IgM	Immunoglobulin M
PC:	Pancreatic cancer
BMI	Body mass index
CP	Chronic pancreatitis
TLR	Toll-like receptor
LAB	Lactic acid bacteria
HCA	Heterocyclic aromatic amines
ALD	Alcoholic liver disease
NAFLD	Nonalcoholic fatty liver disease
NASH	Nonalcoholic steatohepatitis
HSCs	Hepatic stellate cells
TNF	Tumor necrosis factor
TGF-B1	Transforming growth factor B1
IL	Interleukin
NLR	Nod-like receptor
BA	Bile acid
PPI:	Proton pump inhibitor
rRNA	Ribosomal ribonucleic acid
RCT	Randomizede controlled trial
LGG	Lactobacillus rhamnosus
TNF-a	Tumor necrosis factor a
OMT	Oral microbiota transplantation
FMT	Fecal microbiota transplantation
